# The Correlation Between Placental Weight and Neonatal Blood Glucose Levels in Pregnancies With Gestational Diabetes Mellitus

**DOI:** 10.1155/jp/2527672

**Published:** 2025-09-18

**Authors:** Kanthorn Julphakee, Chartrung Patanabapa, Tanchanok Sahassananda, Waranya Pantungthong, Phudit Jatavan, Theera Tongsong

**Affiliations:** Department of Obstetrics and Gynecology, Faculty of Medicine, Chiang Mai University, Chiang Mai, Thailand

**Keywords:** body mass index, gestational diabetes mellitus, maternal weight, neonatal blood glucose, placental weight

## Abstract

**Background:** The primary objective is to identify the correlation between placental weight and neonatal blood glucose levels among pregnancies with gestational diabetes mellitus (GDM). The secondary objectives are to identify the relationships between prepregnancy maternal weight and BMI and placental weight, birth weight and the placental weight-to-birth weight ratio, birth weight and neonatal blood glucose levels, and birth weight and placental weight.

**Methods:** A retrospective cross-sectional study was conducted on GDM patients. The inclusion criteria were a singleton pregnancy, maternal age of 18–40 years, and delivery at term. The obstetric database was accessed to retrieve the consecutive records of GDM for a comprehensive review of the medical records. Maternal and neonatal outcomes, such as placental weight and neonatal blood glucose levels, were collected.

**Results:** A total of 3503 cases were reviewed, and 737 met the inclusion criteria. Placental weight was significantly correlated with prepregnancy maternal weight or BMI, maternal BMI at delivery, and birth weight. The placental weight-to-birth weight ratio was also significantly correlated with prepregnancy maternal weight and birth weight. Placental weight was not significantly correlated with neonatal blood glucose levels, whereas increased birth weight was inversely correlated with neonatal blood glucose levels within 1 h after birth.

**Conclusions:** In women with GDM, placental weight is not significantly correlated with neonatal blood glucose levels, while birth weight is. Birth weight is directly correlated with placental weight. Additionally, prepregnancy maternal weight and BMI are associated with placental weight and the placental weight-to-birth weight ratio.

## 1. Introduction

Gestational diabetes mellitus (GDM) is a common metabolic disorder during pregnancy [1], associated with various complications for fetuses and newborns [2], including fetal macrosomia and neonatal hypoglycemia. These complications are caused by increased fetal insulin production secondary to fetal hyperglycemia. GDM is also linked to placental alterations, such as developmental and anatomical changes, impaired angiogenesis, aberrant villous vascularization, and an imbalance of vasoactive molecules [3]. Additionally, certain placental hormones, such as leptin, which is elevated in women with GDM, can increase placental and fetal size by enhancing protein synthesis and nutrient transport [4, 5]. A large fetus may also have greater oxygen and nutritional demands, potentially causing the placenta to grow larger as a compensatory response [4].

Glucose is transferred through the placenta via facilitated diffusion, meaning that maternal hyperglycemia related to GDM leads to fetal hyperglycemia, which, in turn, stimulates greater fetal insulin production and results in fetal hyperinsulinemia. After birth, placental glucose transfer ceases, but hyperinsulinemia persists, leading to neonatal hypoglycemia [6]. Neonatal hypoglycemia is particularly common in newborns with macrosomia [7].

Several studies have demonstrated a direct relationship between placental weight and birth weight or fetal growth [8–12]. High placental weight *Z*-scores are also associated with adverse neonatal outcomes [13]. Other factors, such as maternal age, maternal weight, prepregnancy BMI [9], maternal hyperglycemia, fasting blood glucose levels [12, 14], and gestational age (GA) [12, 15], can also influence placental weight and birth weight. However, the relationship between placental weight and neonatal blood glucose levels, including neonatal hypoglycemia, has not been previously reported. We hypothesize that, in mothers with GDM, an enlarged placental size which is associated with fetal hyperglycemia, may increase the risk of neonatal hypoglycemia. If this hypothesis is confirmed, placental weight could serve as a clinically relevant prognostic factor in pregnancies affected by GDM. Therefore, this study was primarily conducted to identify the correlation between placental weight and neonatal blood glucose levels in pregnancies with GDM. The secondary objectives were to explore the relationships between (1) prepregnancy maternal weight and BMI and placental weight, (2) birth weight and the placental weight-to-birth weight ratio, (3) birth weight and neonatal blood glucose levels, and (4) birth weight and placental weight.

## 2. Materials and Methods

A retrospective cross-sectional study was conducted on patients with GDM who gave birth at Maharaj Nakorn Chiang Mai Hospital between 2012 and 2021. The study utilized the obstetric database and medical records of the Department of Obstetrics and Gynecology, Chiang Mai University, Thailand. Ethical approval was obtained from the Institutional Review Board (Ethics Committee No. 5, Faculty of Medicine, Chiang Mai University: Study Research ID OBG-2564-08736). The digital obstetric database was accessed, and consecutive records of pregnant women diagnosed with GDM were identified and retrieved. The full medical records of these cases, along with those of their newborns, were comprehensively reviewed and validated for inclusion.

The inclusion criteria were as follows: (1) diagnosis of GDM, (2) maternal age between 18 and 40 years, (3) singleton pregnancy, (4) delivery at term (GA of 37–42 weeks), and (5) availability of complete maternal and neonatal data. The diagnosis of GDM was based on a 100-g 2-h oral glucose tolerance test (OGTT) and was confirmed if at least two of the following thresholds were met or exceeded. The thresholds were fasting glucose ≥ 105 mg/dL, 1-h glucose ≥ 190 mg/dL, 2-h glucose ≥ 165 mg/dL, and 3-h glucose ≥ 145 mg/dL. The exclusion criteria were (1) multifetal pregnancy; (2) smoking; (3) medical conditions such as pregestational DM (Type 2 DM), chronic hypertension, or heart disease; (4) fetal anomalies; (5) preterm birth; and (6) incomplete data.

Baseline characteristics, maternal outcomes, placental outcomes, and neonatal outcomes were reviewed, recorded, and included in the analysis. The primary maternal variables included maternal age, prepregnancy weight, prepregnancy BMI, maternal weight at delivery, and BMI at delivery. Placental outcomes included placental weight and placental diameter. In practice, the placenta, including the umbilical cord but excluding any blood clots, was weighed shortly after birth by the delivering doctor using a standard weighing scale, with the measurement reported in grams. Neonatal outcomes included birth weight and glucose measurements, using the standard Dextrostix (DTX) device, at 1 h after birth, before the first meal, and before the second meal. Additional variables, such as GA at GDM diagnosis, OGTT fasting plasma glucose, OGTT 2-h postload plasma glucose, smoking history, and underlying medical conditions, were also assessed and recorded.

As there had been no prior research on the relationship between placental weight and neonatal blood glucose levels, the sample size was estimated indirectly using previous studies. One study reported a correlation value of 0.673 between placental weight and birth weight among mothers with GDM [4], while another showed a correlation value of 0.210 between GDM and neonatal blood glucose levels [16]. Based on these values, a minimum sample size of 71 pregnant women with GDM was required to achieve a power of 90% at a 95% confidence interval.

### 2.1. Statistical Analysis

All analyses were conducted using SPSS Software Version 26.0 (IBM Corp., Released 2019, IBM SPSS Statistics for Windows, Version 26.0, IBM Corp., Armonk, New York, United States). Baseline data were presented as mean ± standard deviation (SD) or median (interquartile range [IQR]), depending on the data distribution. For comparisons of continuous variables, the independent *T*-test and Mann–Whitney *U* test were used as appropriate. Regression analysis was performed to identify correlations between independent and dependent continuous variables. A *p* value of < 0.05 was considered statistically significant.

## 3. Result

**Figure 1:**
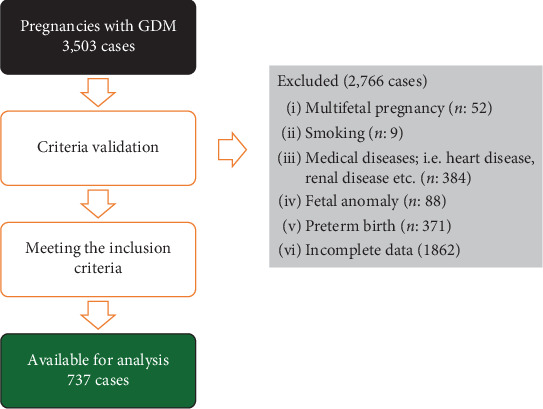
Flowchart of the data recruitment.

During the study period, a total of 3503 pregnant women with GDM gave birth at Maharaj Nakorn Chiang Mai Hospital between 37 and 42 weeks of gestation. Of these, 2766 cases were excluded for various reasons, as shown in Figure 1, leaving 737 cases available for analysis. Among the women included in the analysis, the mean ± SD maternal age was 31.33 ± 4.43 years. The mean ± SD prepregnancy weight and prepregnancy BMI were 58.73 ± 12.70 kg and 23.92 ± 5.37 kg/m^2^, respectively. The mean ± SD birth weight and placental weight were 3137.80 ± 410.64 g and 603.15 ± 132.47 g, respectively. Baseline characteristics of other variables are summarized in Table 1, and the correlations between placental weight and various outcomes are presented in Table 2.

**Figure 2:**
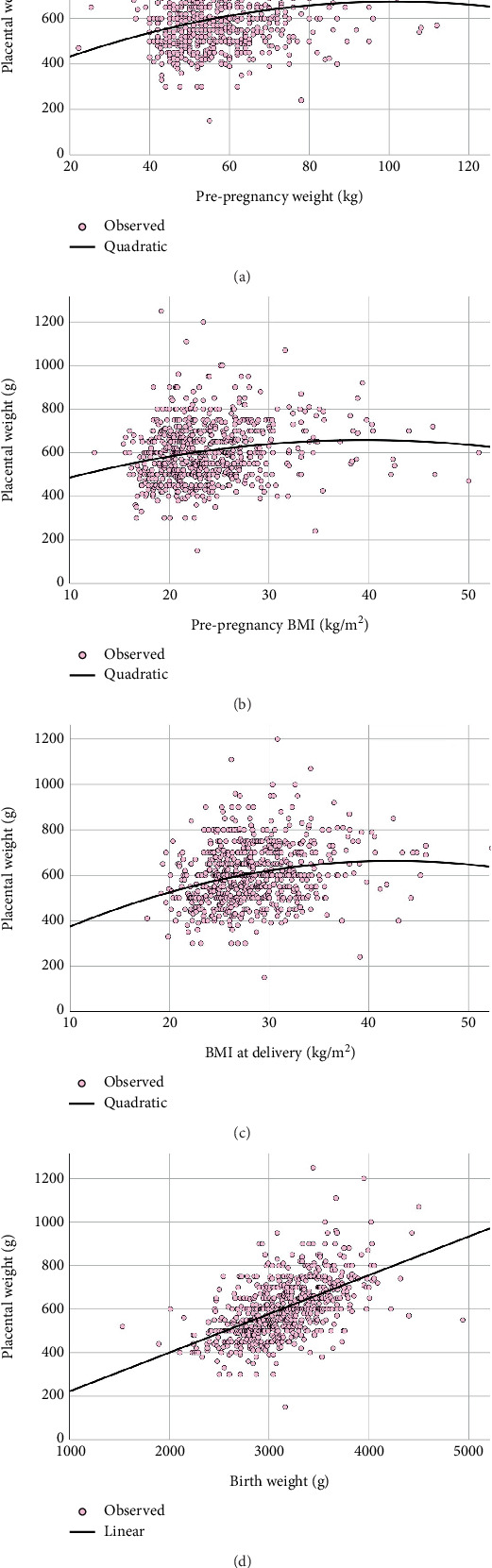
The placental weight was significantly correlated (*p* value < 0.001) with prepregnancy weight (a), prepregnancy BMI (b), and BMI at delivery (c), following a quadratic model, whereas it was significantly correlated (*p* value < 0.001) with birth weight (d), following a linear model.

**Figure 3:**
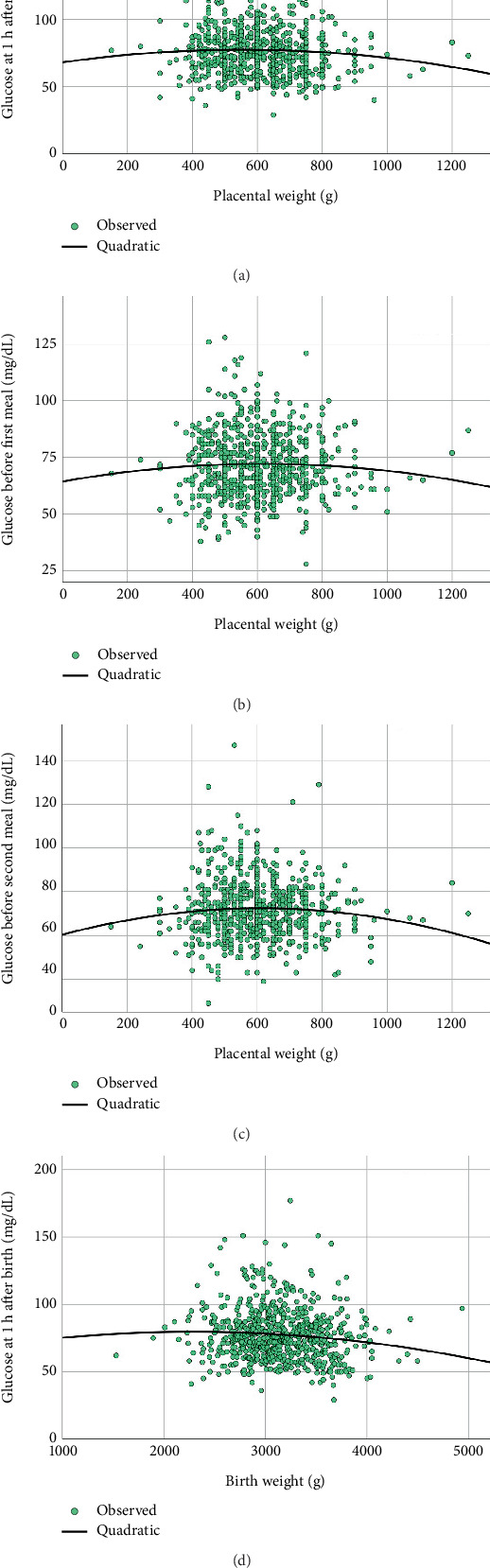
Newborn blood glucose levels at 1 h after birth (a), before the first meal (b), and before the second meal (c) tended to correlate with placental weight following a quadratic model, but this correlation did not reach a significant level (*p* value > 0.05). However, neonatal blood glucose levels at 1 h after birth were significantly correlated (*p* value = 0.008) with birth weight (d) following a quadratic model.

In summary, placental weight significantly increased with higher prepregnancy weight, prepregnancy BMI, BMI at delivery, and birth weight (Figure 2). Similarly, the placental weight-to-birth weight ratio significantly increased with both prepregnancy weight and birth weight. However, neonatal blood glucose levels, represented by DTX measurements within 1 h after birth, before the first meal, and before the second meal, were not significantly associated with either placental weight or the placental weight-to-birth weight ratio (Figure 3). Notably, neonatal blood glucose levels, represented by DTX within 1 h after birth, were inversely and significantly associated with birth weight (Figure 3d).

## 4. Discussion

To the best of our knowledge, this is the first study to examine the correlation between placental weight and neonatal blood glucose levels in pregnancies complicated by GDM. A key insight from this study is that no significant correlation was found between placental weight, or the placental weight-to-birth weight ratio, and neonatal blood glucose levels, as assessed by blood glucose measurements at 1 h after birth, before the first meal, and before the second meal. This study also provides the following confirmatory findings: (1) Birth weight is significantly and inversely correlated with neonatal blood glucose levels. (2) Prepregnancy maternal BMI and weight, as well as maternal BMI at delivery, are directly correlated with birth weight. (3) Prepregnancy maternal BMI and weight, along with maternal BMI at delivery, are directly correlated with placental weight. (4) Placental weight and the placental weight-to-birth weight ratio are significantly and directly associated with birth weight. Contrary to expectations, while an increase in birth weight elevates the risk of neonatal hypoglycemia, an increase in placental weight does not. Therefore, our findings do not support the hypothesis that evaluating placental weight is a useful predictor of neonatal hypoglycemia. The reason why birth weight is more closely associated with neonatal hypoglycemia than placental weight remains unclear. However, it is hypothetically possible that placental size is more strongly correlated with maternal hyperglycemia than with fetal hyperglycemia, the latter of which directly causes neonatal hypoglycemia after the discontinuation of placental glucose transfer at birth. In other words, neonatal hypoglycemia is more directly related to fetal hyperglycemia than to maternal hyperglycemia. Further studies are needed to confirm this hypothesis. Additionally, placental size can be influenced by several factors other than maternal hyperglycemia, such as placental location and placental resistance due to subtle vascular diseases. Therefore, the effect of placental size on neonatal hypoglycemia in patients with GDM, if it exists, could be masked by such confounding factors.

As noted in previous studies [4, 10, 11, 13], this study demonstrated that placental weight had a positive correlation with birth weight. Additionally, both birth weight and placental weight were directly correlated with maternal BMI at delivery among pregnant women with GDM, which is consistent with previous findings [17, 18]. Furthermore, placental weight was significantly associated with prepregnancy maternal BMI, aligning with previous studies [9]. Based on previous research and our findings, placental weight has a strong positive correlation with maternal weight and BMI. It is well known that women with increased prepregnancy weight/BMI are more likely to develop GDM. Accordingly, an increase in placental weight may not be solely caused by GDM; maternal weight and BMI might also play a role. Women with increased BMI prior to pregnancy likely have higher production of leptin, resulting in increased protein synthesis and nutrient transport, which may lead to elevated placental and fetal weights. Gujski et al. [19] reported that the placental weight to fetal weight ratio increased with higher prepregnancy maternal BMI. Although our study found a similar trend, the placental weight to birth weight ratio tended to increase with prepregnancy BMI, though not significantly (*p* value = 0.065). In contrast, it significantly increased with prepregnancy weight, despite the fact that BMI and weight are interrelated. This may be explained by the fact that prepregnancy maternal weight has a more pronounced effect on placental weight than maternal height. The effect appears to diminish when maternal height is taken into account.

The strengths of this study are as follows: (1) The sample size is large enough to address the primary objective of the study. (2) The results could reflect those encountered in actual practice rather than ideal research conditions. (3) The data were extracted and validated through a comprehensive review of the full medical records, not just based on the hospital's crude database.

The weaknesses of this study are as follows: (1) Due to its retrospective nature, medical records may not be entirely reliable, potentially leading to the exclusion of many cases from the analysis. Nevertheless, even after exclusions, the sample size was sufficient to address the primary objective. Additionally, the retrospective nature of the study limited our ability to reliably control for other potential confounding factors affecting newborn glucose levels, such as maternal diet, exercise habits, insulin requirements, and other possible neonatal disorders. (2) Therapeutic interventions could mask the natural course of GDM, meaning the results may primarily reflect well-controlled cases of GDM. However, since the outcomes represent the overall strategy for the diagnosis and management of GDM, the findings reflect real-world practice. Notably, the study still showed a significant effect of birth weight on neonatal hypoglycemia, whereas placental weight did not show such an effect.

In conclusion, this study provides new evidence that, among pregnancies with GDM, placental weight is not significantly associated with neonatal blood glucose levels and is therefore unlikely to be a useful predictor of neonatal hypoglycemia. Additionally, the findings support previous studies, demonstrating that maternal weight and BMI are associated with increases in placental weight, birth weight, and the placental weight-to-birth weight ratio and that birth weight is inversely correlated with neonatal blood glucose levels.

## Figures and Tables

**Table 1:** Baseline characteristics.

	**M** **e** **a** **n** ± **SD**
Age (year)	31.33 ± 4.43
Prepregnancy weight (kg)	58.73 ± 12.70
Prepregnancy BMI	23.92 ± 5.37
Hemoglobin at Lab 1 (g/dL)	12.12 ± 1.18
Hemoglobin at Lab 2 (g/dL)	11.60 ± 1.03
Hematocrit at Lab 1 (%)	36.50 ± 3.45
Hematocrit at Lab 2 (%)	34.99 ± 3.14
BMI at delivery (kg/m^2^)	28.33 ± 4.61
Gestational age at first ANC (week)	9.817 ± 4.61
Gestational age at delivery (week)	38.67 ± 1.13
Birth weight (kg)	3137.80 ± 410.64
Placental weight (kg)	603.15 ± 132.47
Placenta to birth weight ratio	0.19 ± 0.03
Newborn blood glucose within 1 h after birth (mg/dL)	76.98 ± 18.89
Newborn blood glucose before the first meal (mg/dL)	71.84 ± 13.65
Newborn blood glucose before the second meal (mg/dL)	71.86 ± 12.94

Abbreviations: ANC, antenatal care; BMI, body mass index; Lab, laboratory.

**Table 2:** The correlations between various parameters.

**Correlation between**	**Coefficient of determination (** **R** ^2^ **)**	**p** ** value**
Placenta weight (g) and newborn blood glucose (mg/dL) at 1 h after birth	0.003	0.350
Placenta weight (g) and newborn blood glucose (mg/dL) before the first meal	0.004	0.208
Placenta weight (g) and blood glucose (mg/dL) before the second meal	0.008	0.116
Placenta weight (g) and prepregnancy maternal weight (kg)	0.067	< 0.001⁣^∗^
Placenta weight (g) and prepregnancy maternal BMI (kg/m^2^)	0.039	< 0.001⁣^∗^
Placenta weight (g) and maternal BMI (kg/m^2^) at delivery	0.059	< 0.001⁣^∗^
Placenta weight (g) and birth weight (g)	0.303	< 0.001⁣^∗^
Placenta weight (g) to birth weight (g) ratio and newborn blood glucose (mg/dL) at 1 h after birth	0.001	0.412
Placenta weight (g) to birth weight (g) ratio and newborn blood glucose (mg/dL) before the first meal	0.001	0.541
Placenta weight (g) to birth weight (g) ratio and newborn blood glucose (mg/dL) before the second meal	0.005	0.057
Placenta weight (g) to birth weight (g) ratio and prepregnancy maternal weight (kg)	0.017	0.003⁣^∗^
Placenta weight (g) to birth weight (g) ratio and prepregnancy maternal BMI (kg/m^2^)	0.008	0.065
Placenta weight (g) to birth weight (g) ratio and maternal BMI (kg/m^2^) at delivery	0.006	0.149
Placenta weight (g) to birth weight (g) ratio and birth weight	0.039	< 0.001⁣^∗^
Birth weight (g) and newborn blood glucose (mg/dL) at 1 h after birth	0.010	0.008⁣^∗^

Abbreviation: BMI, body mass index.

⁣^∗^*p* value: statistically significant.

## Data Availability

The data that support the findings of this study are available from the corresponding author upon reasonable request.
